# Evaluating the effectiveness of a smartphone app to reduce excessive alcohol consumption: protocol for a factorial randomised control trial

**DOI:** 10.1186/s12889-016-3140-8

**Published:** 2016-07-08

**Authors:** Claire Garnett, David Crane, Susan Michie, Robert West, Jamie Brown

**Affiliations:** Department of Clinical, Educational and Health Psychology, University College London, 1 -19 Torrington Place, London, WC1E 7HB UK; National Centre for Smoking Cessation and Training, London, UK; Cancer Research UK Health Behaviour Research Centre, University College London, London, UK

**Keywords:** Alcohol, Excessive, RCT, App, Smartphone, BCT, Digital, Drink, Digital, Intervention

## Abstract

**Background:**

Excessive alcohol consumption is a leading cause of death and morbidity worldwide and interventions to help people reduce their consumption are needed. Interventions delivered by smartphone apps have the potential to help harmful and hazardous drinkers reduce their consumption of alcohol. However, there has been little evaluation of the effectiveness of existing smartphone interventions.

A systematic review, amongst other methodologies, identified promising modular content that could be delivered by an app: self-monitoring and feedback; action planning; normative feedback; cognitive bias re-training; and identity change. This protocol reports a factorial randomised controlled trial to assess the comparative potential of these five intervention modules to reduce excessive alcohol consumption.

**Methods:**

A between-subject factorial randomised controlled trial. Hazardous and harmful drinkers aged 18 or over who are making a serious attempt to reduce their drinking will be randomised to one of 32 (2^5^) experimental conditions after downloading the ‘Drink Less’ app. Participants complete baseline measures on downloading the app and are contacted after 1-month with a follow-up questionnaire. The primary outcome measure is change in past week consumption of alcohol. Secondary outcome measures are change in AUDIT score, app usage data and usability ratings for the app. A factorial between-subjects ANOVA will be conducted to assess main and interactive effects of the five intervention modules for the primary and secondary outcome measures.

**Discussion:**

This study will establish the extent to which the five intervention modules offered in this app can help reduce hazardous and harmful drinking. This is the first step in optimising and understanding what component parts of an app could help to reduce excessive alcohol consumption. The findings from this study will be used to inform the content of a future integrated treatment app and evaluated against a minimal control in a definitive randomised control trial with long-term outcomes.

**Trial registration:**

ISRCTN40104069 Date of registration: 10/2/2016

**Electronic supplementary material:**

The online version of this article (doi:10.1186/s12889-016-3140-8) contains supplementary material, which is available to authorized users.

## Background

Excessive alcohol consumption is responsible for approximately 3.3 million deaths each year worldwide [1]; only high blood pressure and smoking contribute more to the global burden of disease [2]. When the impact of alcohol-related crime and lost productivity is added to healthcare, alcohol consumption costs the UK economy an estimated £21bn per year [3]. Tackling excessive alcohol consumption is a public health priority [4] and there is a need for interventions to help people reduce their consumption.

In the UK, although brief interventions for excessive alcohol use are available and appear to be both effective [5] and cost-effective [6, 7], they are not widely offered; less than 10 % of those drinking excessively receive a brief intervention on alcohol from their general practitioner (GP) [8]. Digital behaviour change interventions (DBCIs) delivered on websites, by email or through mobile phones offer the potential to increase the proportion of excessive drinkers receiving an alcohol brief intervention [9]. The convenience and anonymity of DBCIs may increase uptake amongst those reluctant to receive help from health professionals [10, 11]. DBCIs have been found in meta-analyses and systematic reviews to result in small reductions in alcohol consumption across a range of populations [12–20]. A 2016 Cochrane review of 40 RCTs found that DBCIs reduced alcohol consumption by 23.6 g of alcohol per week (equivalent of 2.95 UK units) more than controls [21, 22].

The rapid development and use of health-related smartphone apps provides a new method for supporting people in their attempts to reduce their alcohol consumption. It is estimated that the 165,000 currently available smartphone apps for the practice of medicine and public health [23] will be downloaded a total of three billion times in 2015, almost double the number from 2013 [24]. Despite their proliferation, evaluation of app content has revealed they are often developed without reference to scientific evidence or theory, fail to conform to guidelines, lack evidence based-content and/or provide inaccurate information [25–31]. There has been little evaluation of app effectiveness. Reviews of mobile phone interventions to promote weight loss [32, 33], improve women’s health [34], increase physical activity [35] and improve treatment adherence for chronic disease management [36], as well as a review of digital resources for mental health self-management [37], found numerous trials of text messaging but few RCTs of apps. When they have been evaluated, apps have generally been found effective. Apps have increased physical activity [38], improved muscular fitness, movement skills, and weight-related behaviours [39], reduced symptoms of depression [40, 41] and improved diabetes management [42]. Only one trial of alcohol reduction apps appears to have been published; this evaluated two different apps aimed at Swedish university students, both allowed users to calculate their levels of blood alcohol concentration, though neither was effective at reducing consumption relative to controls [43].

The problem of excessive alcohol consumption and the potential of smartphone apps to help people manage their behaviour, as well as the limited evidence for the effectiveness of such apps and their tendency to be developed without reference to scientific evidence or theory, highlight the need for the rigorous development and evaluation of new smartphone app alcohol interventions.

### Selecting modules for evaluation

The initial selection of modules for evaluation was based on four main sources of evidence: i) examination of the behaviour change techniques (BCTs) used in alcohol interventions [44]; ii) a systematic review of the evidence of the effectiveness of digital technologies for reducing excessive alcohol consumption [21, 22]; iii), a formal consensus-building study with experts in the fields of alcohol or behaviour change to identify the behaviour change techniques thought most likely to be effective at reducing alcohol consumption in a smartphone app [45]; iv), a content analysis of the behaviour change techniques within existing popular alcohol reduction apps [46]. On the basis of this systematic development work, the following five modules were selected as high priority for experimental manipulation evaluation in a factorial design: self-monitoring and feedback; action planning; normative feedback; cognitive bias re-training; and identity change. We elaborate the reasons for each selection below.

### Self-monitoring and feedback

Self-monitoring and feedback are both recommended as effective techniques for alcohol reduction by the National Institute for Health and Care Excellence (NICE) clinical guidance [47]. They are also related to core elements of Control Theory [48], which posits that behaviour is goal-driven and that feedback enables people to assess their performance in relation to their goals and make adjustments toward it accordingly. Self-monitoring has been found to be effective for controlling weight and blood-glucose levels [49–52]; increasing academic performance [53, 54] and improving healthy eating and physical activity [55]. In the formal consensus-building study with behaviour change or alcohol experts, self-monitoring was ranked the most likely intervention component to be effective in a smartphone app to reduce excessive alcohol consumption [45]. Behaviour change interventions which include self-monitoring in combination with at least one of the other behaviour change techniques (BCTs) relevant to Control Theory have been found to be significantly more effective than interventions not including those techniques [55–57]. Feedback is a key component of brief alcohol interventions [58] and is commonly included in DBCIs: 95 % of the DBCIs in the 2016 Cochrane review gave participants feedback about their drinking [59]. Feedback was also ranked highly as an intervention component likely to be effective in an app to reduce excessive alcohol consumption by alcohol or behaviour change experts in a formal consensus-building study [45].

### Action planning

NICE clinical guidance recommends that providers of behaviour change interventions for alcohol reduction should facilitate action planning [47]. Action planning is also a technique related to a core element of Control Theory, reducing discrepancies between goals and observed behaviour [48]. Action plans detailing the steps necessary to achieve a specific goal have been found to increase physical activity [60], enhance behaviour change in patients [61] and reduce alcohol consumption [62–64]. ‘Implementation intentions’, a form of action plan that enable the setting of if/then conditions for future events [65], increased goal-attainment rates for health behaviours such as regular breast examinations [66], engaging in exercise [67] and alcohol reduction [62–64].

### Normative feedback

Normative feedback is personalised feedback on how an individual’s behaviour compares with the behaviour of other people. Providing normative feedback can reduce subsequent alcohol use [68–73] indicating that normative misperceptions (underestimating own alcohol use compared with others) play a role in excessive alcohol consumption. Research has shown that normative misperceptions exist in the general population [74] as well as in heavy drinkers [69, 70] and college/university students [71, 72, 75–77]. Theoretical evidence for the role of normative misperceptions in excessive alcohol consumption come from Social Norms theory [78]. This theory predicts that people behave in a way that attempts to conform to the perceived norm. This can result in people behaving in ways that are not consistent with their own beliefs and values in their attempt to reach the perceived norm [79]. Providing feedback in relation to people was also identified by alcohol or behaviour change experts as an intervention component likely to be effective at reducing excessive alcohol consumption in a smartphone app [45].

### Cognitive bias re-training

Dual process theories of addiction [80–82] suggest that excessive alcohol consumption occurs, in part, due to automatic processes when the impulses to drink overcome the inhibitory response not to [83]. These automatic biases in information processing of alcohol-related cues or stimuli have been found to predict alcohol use [84, 85] though are largely unaffected by interventions targeting changing conscious information or processes [86, 87]. Cognitive bias re-training has been found to be effective at altering these automatic cognitive biases [88–92] and some studies have also found there are associated impacts on subsequent alcohol use [90, 91, 93, 94]. The intervention strategy chosen for this module is to re-train approach biases, with the aim of changing the tendency to approach alcohol and alcohol-related stimuli to an ‘avoid’ bias. Retraining these approach biases has been shown to have a greater efficacy in reducing alcohol consumption [90–92] than retraining other cognitive biases such as attentional biases [95].

### Identity change

Excessive drinking is central to many peoples’ sense of self, particularly students [96], and identity has been proposed as a motivational factor for behaviours by a number of theories [97–99], including the PRIME theory of motivation which proposes that identity is a source of motives, self-regulation and stability of behaviour [100]. Identity (group, social and/or individual) was also identified in a consensus approach as a theoretical domain to explain behaviour change [101]. The relationship between identity and behaviour change has not been investigated in the field of alcohol research though there is evidence from the smoking cessation literature that identity change (adopting an identity that is incongruent with the undesired behaviour) may be an effective intervention technique. A systematic analysis of English Stop Smoking Services treatment manuals found that ‘strengthening an ex-smoker identity’ was associated with 4-week abstinence rates (both carbon-monoxide verified and self-reported) [102]. A positive smoker identity was present in a minority of smokers in England and predicted failure to make a smoking quit attempt at 6 months and so may be an important barrier to behaviour change [103]. A meta-ethnography also found that the nature of a smoker’s identity can play an important role in smoking cessation [104].

Each of the intervention modules detailed above contain a number of relevant behaviour change techniques, details of the full content of each module are summarised in Additional file 1: Table S1. To evaluate both the overall effectiveness of the app and its component modules, we will use a full factorial study design, guided by the Multiphase Optimization Strategy [105]. This uses factorial experiments to screen possible intervention components selected on the basis of theory and evidence to identify those warranting further investigation [106], with users randomly allocated to receive either an enhanced (‘high’) or minimal (‘low’) version of each intervention module.

## Methods

### Aim

The aim of the study is to evaluate the effectiveness of five intervention modules at reducing excessive alcohol consumption.

### Design

A between-subject factorial randomised controlled trial evaluating the effectiveness of five intervention modules (i) self-monitoring and feedback, ii) action planning, iii) normative feedback, iv) cognitive bias re-training, and v) identity change), all with a ‘high’ and ‘low’ version (see Additional file 1: Table S1) yielding 32 experimental conditions (see Additional file 2: Table S2). This factorial design was chosen over a treatment package approach with usual care or nothing as a control group so that the individual effect of each intervention module on excessive alcohol consumption can be assessed. A factorial design also requires smaller sample sizes than individual experimental designs whilst still maintaining the same power.

### Intervention

Drink Less is an app available for iOS devices that is designed to support a user who is interested in cutting down their alcohol consumption. The iOS (Apple’s operating system) was chosen to avoid issues of fragmentation associated with Android [107] and because there tends to be a greater retention rate for apps amongst iPhone users compared with Android [108]. There was a pragmatic, methodological need to structure the app around an activity that would engage all users and allow experimental manipulation of other supporting modules. Thus, the app asks all users to set a goal to which they would like to reduce their alcohol consumption. The app then offers them access to a variety of modules and tools to help them achieve their goal. The app was interactive though there was no human component to its functionality. The content of these five intervention modules is described in detail in Additional file 1: Table S1. The ‘high’ version of each intervention module contained the BCTs or intervention component hypothesised to be effective. The ‘low’ version of each intervention module lacked the BCTs or intervention component being assessed and, where possible, were based on controls in equivalent studies.

### Study sample

Participants will be included in the analysis if they have downloaded the app onto an iOS smartphone or tablet, are 18 years of age or over, live in the United Kingdom and have an AUDIT score of 8 or above (indicative of excessive alcohol consumption), have confirmed that they are making an attempt to reduce their drinking (responded “Interested in drinking less alcohol”, not “Just browsing” to “Why are you using this app?”), and provided an email address.

This study will recruit 672 participants and have more than 80 % power (with alpha at 5 %, 1:1 allocation and a two-tailed test) to detect a mean change in alcohol consumption of 5 units between the high and low condition for each intervention module [109]. This assumes a mean of 27 weekly units at follow-up in the control group, a mean of 22 units in the intervention group and a SD of 23 units for both (d = 0.22), and rounds up the sample size to the nearest multiple of 32 to ensure all cells are balanced. The estimated effect size is large (comparable with that of a face-to-face brief intervention [5]) and may be considered somewhat unrealistic for a module within a digital intervention. However, in the event of a ‘non-significant’ result, we plan to calculate a Bayes factor to establish the relative likelihood of the null versus the experimental hypothesis given the data obtained [110]. This will permit a relative judgment for the purposes of screening about whether the inclusion of the module in a future app would be more likely than not to have an effect on alcohol consumption.

### Recruitment

Participants will be recruited through a number of methods. The app will be listed in the iTunes Store and the listing will be optimised according to best practices for app store optimisation (e.g. ensuring the keywords are carefully selected, that the description is well written and that screenshots display the aspects of the app that users are most interested in [111–114]). Users will be encouraged to leave reviews, which may persuade others to download it [114, 115]. We intend to promote the app through organisations such as the Department of Health and Public Health England, and mHealth (mobile health) directory web sites (e.g. ourmobilehealth.co.uk, myhealthapps.net), alcohol-reduction online forums (e.g. Club Soda) and the UCL App Lab service that promotes apps to all of the staff and students at UCL.

### Procedure

Each participant, on downloading the app, will be asked to read the participant information sheet and provide informed consent. Before being able to access the content of the app participants are asked to provide socio-demographic data, indicate their reason for using the app (interested in drinking less alcohol or just browsing), provide their e-mail address for the 1-month follow-up questionnaire and complete the full AUDIT questionnaire. At this point, all participants who meet the inclusion criteria will be randomised to one of 32 unique experimental conditions (see Additional file 2: Table S2) in a block randomisation method. After this they are provided with their AUDIT score and informed of their ‘AUDIT risk zone’. From this point onwards, the app differs for the different experimental conditions. Participants who do not meet all of the inclusion criteria can still use the app and will be allocated to a separate, non-experimental condition that has the ‘high’ version of each intervention module for engagement and app rating purposes.

One month after downloading the app, the app will automatically deliver a follow-up questionnaire. If this is not completed, email reminders will be sent at periodic intervals (1 day and 1 week). The follow-up questionnaire consists of the AUDIT and questions regarding usability.

### Measures

#### Baseline measures

AUDIT score; socio-demographic assessment (age, gender, ethnicity, level of education, employment status and whether they are a current smoker).

### Outcome measures

The primary outcome measure is change in past week consumption of alcohol; calculated from the AUDIT-C score at baseline and 1-month follow-up [109]. Secondary outcome measures will be i) change between baseline and follow-up on the full AUDIT score ii) app usage data (user sessions per day, screen views per day, screens per session, session duration and session instances, user retention), and iii) usability ratings for the app (a) how helpful did you find Drink Less? b) how easy did you find Drink Less to use? c) how satisfied are you with Drink Less? d) how likely are you to recommend Drink Less to a friend?). An intention-to-treat approach will be used such that those who are lost to follow-up will be retained in the primary analysis and assumed to be drinking at baseline levels. The full 10-item AUDIT assesses alcohol consumption (AUDIT-C), harmful drinking and alcohol dependence [116]. The AUDIT has been used in other trials for assessing alcohol consumption and related harms [117].

### Analysis

A factorial between-subjects ANOVA will be conducted to assess main and interactive effects of the five intervention modules on the primary and secondary outcomes. In a sensitivity analysis, ANCOVAs will also be conducted to adjust for any chance imbalances in drinking and socio-demographic characteristics (gender, age, ethnicity, level of education, employment status, AUDIT score, AUDIT-C score).

On the basis of the intention-to-treat principle, individuals who are not followed up (non-responders) will be retained in the analyses and assumed they drinking at same levels as baseline. Sensitivity analyses will be conducted i) among only those who completed the follow-up questionnaire (responders) and ii) by imputing missing data from baseline characteristics. The intention-to-treat principle is often used in digital public health interventions [118–120] and is a conservative approach to ensure effect sizes are not over-estimated as participants who respond well to the intervention are more likely to be retained.

In the event of a non-significant main effect of an intervention module, Bayes factors will be calculated with the alternative hypotheses conservatively represented in each case by a half-normal distribution (online calculator: http://www.lifesci.sussex.ac.uk/home/Zoltan_Dienes/inference/Bayes.htm). In an alternative hypothesis represented by a half-normal distribution, the standard deviation of a distribution can be specified as an expected effect size, which means plausible values have been effectively represented between zero and twice the effect size, with smaller values more likely. The expected effect size for the primary calculation of Bayes factors will be the same as for the power calculation (d = 0.22). In a sensitivity analysis, we will also calculate Bayes factors for a smaller effect (reflecting a reduction of 3 units per week, d = 0.13).

## Discussion

This study protocol describes the design of a factorial randomised controlled trial to determine the effectiveness of five intervention modules delivered within a smartphone app at reducing excessive alcohol consumption. To our knowledge, this will be the first study to examine the effectiveness of a smartphone app to reduce excessive alcohol consumption that has been developed based on empirical evidence and theoretical models.

This type of trial and analysis means we can independently assess each module to see which module is having the biggest effect. This also allows for on-going evaluation and optimisation of the app for future evaluation of an integrated treatment package. As each module was developed based on empirical evidence and theoretical models, the findings of this study will be able to inform behavioural science, theory and future public health interventions. The ‘pure control’ group in this trial was effectively those who received ‘low’ versions of every intervention module which lacked the BCTs or intervention component being assessed in the ‘high’ version. Most popular alcohol reduction apps include almost no BCTs or mentions of theory [46], therefore the users receiving the ‘pure control’ were effectively receiving ‘usual care’ in this context.

The selection process of high priority modules for evaluation was intended to be systematic and transparent. However, it is possible that other researchers could have conducted a similar process and reached a different view. For example, it may have been of interest to evaluate the individual effect of goal-setting. Goal-setting was provided to all participants for two reasons. First, from a methodological perspective, we believed there was a pragmatic need to provide engaging content to all users, particularly those receiving low versions of all the modules, and around which access to other modules could be plausibly structured. Second, we thought the evidence-base on goal-setting was sufficiently robust that it would warrant inclusion in a future evaluation of an integrated app without support from a factorial screening experiment [121–123].

Our power calculation relied on a large estimated effect size (comparable with that of a face-to-face brief intervention) and may be considered somewhat unrealistic for a module within a digital intervention. The reason is that selecting smaller effect sizes would require larger numbers of participants and more time to recruit them. The Multiphase Optimization Strategy, which guides our research, emphasises agile screening experiments before running a definitive head-to-head trial of an optimised intervention against a control [106, 124]. We deal with the limitation of a somewhat unrealistic effect size for our power calculation by planning to supplement our inferential statistics by calculating Bayes factors. Bayes factors will provide useful information on the relative likelihood of smaller (more realistic) effects compared with the null given the data we obtain.

One strength of this intervention is that it is delivered by smartphone, so there will be no issues of availability or accessibility for the participants. The app can be used fully without an internet connection. The data is stored on the phone until an internet connection is available, when the data is then sent to the server. A limitation of this type of study is high attrition. We will send regular reminders for the one-month follow-up questionnaire and remind them of the incentives offered to reduce the risk of attrition. A practical issue may be recruiting enough participants to meet the numbers sufficient to meet the power for our analysis. We have planned for this issue by using best practices for app store optimisation and by promoting the app through trusted organisations such as Public Health England and University College London.

This study will evaluate the extent to which an app containing five intervention modules (self-monitoring combined with feedback, action planning, normative feedback, cognitive bias re-training, and identity change), developed based on theory and empirical evidence, can help reduce excessive alcohol consumption. Each intervention module will be independently assessed and the findings will be used to inform the content of a future app with an integrated treatment package that will be evaluated against a minimal control in a definitive randomised control trial with long-term outcomes. As the app and its intervention modules have been developed based on theoretical models and empirical evidence, these findings will also be able to inform future behaviour change interventions, theories and behavioural science.

## Abbreviations

BCT, behaviour change technique; DBCI, digital behaviour change intervention; RCT, randomised controlled trial.

## Additional files

Additional file 1: RCT protocol Additional file 1. **Table S1.** Details of intervention modules. Table detailing the full content of the five intervention modules, both the ‘high’ and ‘low’ versions. (DOCX 19 kb) Additional file 2: RCT protocol Additional file 2. **Table S2.** Experimental group matrix. Table showing the 32 experimental conditions. (DOCX 56 kb)
