# Supplementary material for: Evaluating the effectiveness of a smartphone app to reduce excessive alcohol consumption: protocol for a factorial randomised control trial
Source: BMC Public Health. 2016 Jul 8;16:536. doi: 10.1186/s12889-016-3140-8 (PMC4939028; doi:10.1186/s12889-016-3140-8)
Supplement: Additional file 1: — RCT protocol Additional file 1. Table S1. Details of intervention modules. Table detailing the full content of the five intervention modules, both the ‘high’ and ‘low’ versions. (DOCX 19 kb) [file 12889_2016_3140_MOESM1_ESM.docx]

Additional file 1: Table S1: Details of intervention modules

| Intervention module | Objective | Details of module | | BCTs included in ‘high’ version | BCTs included in ‘low’ version |
| --- | --- | --- | --- | --- | --- |
|  |  | High | Low |  |  |
| Self-monitoring and feedback | Facilitate easy and on-going recording of alcohol consumption; provide feedback on consumption, consequences of consumption and progress toward goals. | Ability to record drinks, graph showing units consumed calories consumed, amount spent on alcohol. Record mood, productivity, clarity, sleep quality, graph illustrating how they differ on mornings after heavy drinking compared to mornings after light/no drinking. Feedback on progress towards goals: cumulatively as the week progresses, on the past week and on all previous weeks. | Ability to record drinks. No other self-monitoring facilitated. No feedback provided | Review behaviour goals, Discrepancy between current behaviour and goal, Feedback on behaviour, Self-monitoring of behaviour, Self-monitoring of outcomes of behaviour, Feedback on outcomes of behaviour, Salience of consequences, Information about emotional consequences, Social reward, Self-reward | Self-monitoring of behaviour |
| Action planning | Allow users to create implementation intentions for dealing with difficult drinking situations. | Create implementation intentions, review implementation intentions already created, gain understanding of why to set implementation intentions. | Gain understanding of why to set implementation intentions only. | Action planning,  Credible source | None |
| Normative feedback | Inform users of the social drinking norm and alert them to any discrepancy with how they believe their drinking compares with normal to how it actually compares with normal. | Questions assessing how users think they compare with others.  Infographics illustrating how user’s drinking actually compares with other adults and others of same gender and age. | Text on risks of drinking too much (from PHE website) | Social comparison  Provide normative information about others’ behaviour and experiences | Information about health consequences  Information about emotional consequences |
| Identity change | Help users foster a change in their identity so that users do not see themselves as “drinkers” as a key part of their identity which should aid their behaviour change attempt. | Memos – record messages about drinking or when drunk too much to watch in the future  “I am”: identifying and considering those values that are important to you, and whether you do not live up to those values when you have drunk too much.  Flipsides of drinking:  Providing examples of the negative sides of positive alcohol expectancies. | Text on how identity is an important factor in how we behave and advise to think about the undesired consequences of drinking too much. | Identification of self as role model  Incompatible beliefs  Valued self-identity  Identity associated with changed behaviour  Information about health/social/emotional consequences  Salience of consequences  Anticipated regret  Pros and cons  Framing/reframing | None |
| Cognitive bias re-training | Use a form of cognitive bias modification to strengthen cognitive control over the automatic biases to approach alcohol that predict alcohol use that exist amongst the users through an engaging game. | Game with all alcohol related pictures associated with “avoid” and all soft drink pictures associated with “approach”.  Additional section of text on why and how this sort of game is believed to work. | Game with 50% of alcohol related pictures associated with “avoid” and 50% associated with “approach”. Same for soft drink pictures. | None | None |
