# Supplementary material for: Evaluating the effectiveness of a smartphone app to reduce excessive alcohol consumption: protocol for a factorial randomised control trial
Source: BMC Public Health. 2016 Jul 8;16:536. doi: 10.1186/s12889-016-3140-8 (PMC4939028; doi:10.1186/s12889-016-3140-8)
Supplement: Additional file 2: — RCT protocol Additional file 2. Table S2. Experimental group matrix. Table showing the 32 experimental conditions. (DOCX 56 kb) [file 12889_2016_3140_MOESM2_ESM.docx]

Additional file 2: Table S2: Experimental group matrix (H: ‘high’; L: ‘low’)

| Experimental group | Normative feedback | Identity change | Cognitive bias re-training | Self-monitoring & feedback | Action planning |
| --- | --- | --- | --- | --- | --- |
| 1 | H | H | H | H | H |
| 2 | H | H | H | H | L |
| 3 | H | H | H | L | H |
| 4 | H | H | H | L | L |
| 5 | H | H | L | H | H |
| 6 | H | H | L | H | L |
| 7 | H | H | L | L | H |
| 8 | H | H | L | L | L |
| 9 | H | L | H | H | H |
| 10 | H | L | H | H | L |
| 11 | H | L | H | L | H |
| 12 | H | L | H | L | L |
| 13 | H | L | L | H | H |
| 14 | H | L | L | H | L |
| 15 | H | L | L | L | H |
| 16 | H | L | L | L | L |
| 17 | L | H | H | H | H |
| 18 | L | H | H | H | L |
| 19 | L | H | H | L | H |
| 20 | L | H | H | L | L |
| 21 | L | H | L | H | H |
| 22 | L | H | L | H | L |
| 23 | L | H | L | L | H |
| 24 | L | H | L | L | L |
| 25 | L | L | H | H | H |
| 26 | L | L | H | H | L |
| 27 | L | L | H | L | H |
| 28 | L | L | H | L | L |
| 29 | L | L | L | H | H |
| 30 | L | L | L | H | L |
| 31 | L | L | L | L | H |
| 32 | L | L | L | L | L |
